# Distribution and prevalence of musculoskeletal pain co-occurring with persistent low back pain: a systematic review

**DOI:** 10.1186/s12891-020-03893-z

**Published:** 2021-01-18

**Authors:** Cecilie K. Øverås, Melker S. Johansson, Tarcisio F. de Campos, Manuela L. Ferreira, Bård Natvig, Paul J. Mork, Jan Hartvigsen

**Affiliations:** 1 Department of Sports Science and Clinical Biomechanics, Faculty of Health Sciences, University of Southern Denmark, Odense, Denmark; 2 Department of Public Health and Nursing, NTNU - Norwegian University of Science and Technology, Trondheim, Norway; 3 Department of Health Professions, Faculty of Medicine, Health and Human Sciences, Macquarie University, Sydney, NSW Australia; 4 Faculty of Medicine and Health, Institute of Bone and Joint Research, The Kolling Institute, Northern Clinical School, The University of Sydney, Sydney, NSW Australia; 5 Department of General Practice, Institute for Health and Society, University of Oslo, Oslo, Norway; 6 Nordic Institute of Chiropractic and Clinical Biomechanics, Odense, Denmark

**Keywords:** Low back pain, Comorbidity, Musculoskeletal pain, Prevalence, Systematic review

## Abstract

**Background:**

Co-occurring musculoskeletal pain is common among people with persistent low back pain (LBP) and associated with more negative consequences than LBP alone. The distribution and prevalence of musculoskeletal pain co-occurring with persistent LBP has not been systematically described, which hence was the aim of this review.

**Methods:**

Literature searches were performed in MEDLINE, Embase, CINAHL and Scopus. We considered observational studies from clinical settings or based on cohorts of the general or working populations involving adults 18 years or older with persistent LBP (≥4 wks) and co-occurring musculoskeletal pain for eligibility. Study selection, data extraction and risk of bias assessment were carried out by independent reviewers. Results are presented according to study population, distribution and location(s) of co-occurring pain.

**Results:**

Nineteen studies out of 5744 unique records met the inclusion criteria. Studies were from high-income countries in Europe, USA and Japan. A total of 34,492 people with persistent LBP were included in our evidence synthesis. Methods for assessing and categorizing co-occurring pain varied considerably between studies, but based on the available data from observational studies, we identified three main categories of co-occurring pain – these were axial pain (18 to 58%), extremity pain (6 to 50%), and multi-site musculoskeletal pain (10 to 89%). Persistent LBP with co-occurring pain was reported more often by females than males, and co-occurring pain was reported more often in patients with more disability.

**Conclusions:**

People with persistent LBP often report co-occurring neck pain, extremity pain or multi-site pain. Assessment of co-occurring pain alongside persistent LBP vary considerable between studies and there is a need for harmonisation of measurement methods to advance our understanding of how pain in different body regions occur alongside persistent LBP.

**Systematic review registration:**

PROSPERO CRD42017068807.

**Supplementary Information:**

The online version contains supplementary material available at 10.1186/s12891-020-03893-z.

## Background

Low back pain (LBP) is common and a worldwide leading and growing cause of disability with enormous costs [1]. For some people, due to multifactorial reasons [1], LBP persists and becomes a long-lasting condition [2, 3]. Individuals with persistent LBP commonly presents with a range of additional health problems and diseases such as sleep disorders, anxiety and depression [4–7], as well as co-occurring musculoskeletal pain [8–11]. Musculoskeletal pain in other body sites is also found to be significantly associated with new-onset LBP [12].

Persistent LBP with co-occurring musculoskeletal pain is reported to be more frequent and distinctly different from persistent LBP that occur alone [13–15]. The presence of co-occurring musculoskeletal pain is associated with poor prognosis, more negative health outcomes, and increased health care utilization [5, 16–20]. Persons with LBP have increased likelihood of co-occurring pain elsewhere in the spine [8], but LBP has also been found to cluster with lower extremity pain [21]. Yet, the current scientific literature on persistent LBP lack an overview of the distribution and prevalence of co-occurring pain sites.

Persistent LBP is often treated as a condition on its own – irrespective of musculoskeletal comorbidity, but may need to be viewed beyond just a regional pain site problem [9]. Despite common prognostic factors across different musculoskeletal pain sites [22], different LBP phenotypes (i.e., different definitions of LBP), may have different prognoses and benefit from different management approaches. To improve patient outcomes, it is important to identify these LBP phenotypes and get a better insight into the prevalence of this multifaceted and complex problem.

The overall aim of this systematic review was therefore to critically appraise and summarize the literature dealing with the distribution and prevalence of co-occurring musculoskeletal pain among people with persistent LBP. The research questions addressed were: (1) What are the patterns of distribution of co-occurring musculoskeletal pain (i.e., number of co-occurring pain sites, distribution across body quadrants, combination of sites and general pattern) among people with persistent LBP? (2) What is the prevalence of co-occurring musculoskeletal pain among people with persistent LBP? (3) Is there an association between pain patterns and/or number of pain sites and age, sex, or LBP-related disability?

## Methods

The protocol for this review was prospectively registered in the PROSPERO database (CRD42017068807) and published [23]. This systematic review was reported following the Preferred Reporting Items for Systematic Reviews and Meta-Analysis (PRISMA) Statement [24] (Additional file 1).

### Eligibility criteria

We considered observational studies (i.e., longitudinal and cross-sectional cohort studies), from clinical settings or based on cohorts of the general or working populations involving adults 18 years or older. We included studies investigating persistent LBP (e.g., pain within the anatomical region below the twelfth thoracic vertebra and the inferior gluteal fold), with or without radiation to the legs, with a duration of at least 4 wks. Furthermore, eligible studies had to assess co-occurring musculoskeletal pain (i.e., number of co-occurring pain sites and distribution across body sites) in individuals with persistent LBP. Peer-reviewed studies published in the English, Dutch, Danish, Norwegian, Portuguese, Spanish, or Swedish languages, understood by the authors of this paper, were screened for eligibility. Studies including individuals with LBP of specific pathological origin (e.g., fracture, tumour, inflammatory diseases, systemic diseases, infection, structural deformity) were excluded, as were studies including pregnant women and studies dealing with post-surgical persistent LBP. Studies with other study designs (e.g., randomised controlled trials) as well as studies with a study sample of < 100 individuals with persistent LBP were post-protocol decided to be excluded due to their limited possibility to assess prevalence.

### Database search strategy

The literature search was performed with no restrictions on date, publication type, or language within the following bibliographic databases: MEDLINE and Embase (via Ovid), CINAHL, and Scopus (for forward citation tracking), from the earliest records published to August 2nd, 2019. We updated the search on October 26th, 2020 in Medline (via Ovid) only, as there were no unique hits in the other databases and since majority of relevant studies in systematic reviews are reported to be found within a limited number of databases without introducing bias or changing results [25]. Search terms covered the following domains: LBP, co-occurring musculoskeletal pain, and number of pain sites/pain patterns, combined with study design. Pilot searches were performed on the search terminology to ensure its all-inclusiveness. The design and execution of the searches were supervised by a research librarian (see Additional file 2 for the search strategy). The reference lists of included articles and related reviews within the topic were scrutinised, and forward citation tracking was performed on key articles in order to identify any further studies. PROSPERO was inspected for ongoing or recently completed systematic reviews to identify additional articles not identified in the bibliographic databases. We did not search additional grey literature as originally planned as we do not expect to find any relevant epidemiological studies here as opposed to literature on interventions where very few relevant studies or questionable vested interests may have an impact on the results [26]. The identified articles were downloaded to and managed in EndNote X9 [27].

### Study selection

Relevant records were selected through a two-stage screening process by three independent reviewers (CKØ, MSJ and TFC), where one reviewer (CKØ) screened all and the other two (MSJ and TFC) shared the screening of the retrieved records. In the first stage, titles and abstracts were screened with the reviewers blinded to each other’s selections. Disagreements were discussed and resolved by a fourth independent reviewer (BN) if necessary. The studies considered not to be relevant, or that clearly did not meet the inclusion criteria, were excluded, and full-text articles of the remaining studies were obtained. Studies relevant for the topic, but with uncertain relevance for the current review were taken to the second stage for further consideration. In the second stage, the same reviewers made the final selection based on screening of the full text articles against the eligibility criteria. If necessary, study authors were contacted for additional information to resolve questions about eligibility. Consensus meetings were used to resolve any disagreement by consulting a fourth reviewer (JH). Reasons for exclusion were recorded.

### Data extraction

Data from the included articles were extracted into pre-tested forms by two independent author pairs (CKØ + MLF/JH and TFC + MLF/JH), each pair including an experienced reviewer. Disagreements were resolved first by discussion or if necessary by a third independent group of reviewers (MSJ, BN and PJM). Data extraction included: main characteristics of the included articles, definition of LBP, prevalence of co-occurring musculoskeletal pain by anatomical location, number of co-occurring pain sites and/or pattern of distribution of co-occurring musculoskeletal pain, association between the pain pattern and/or number of pain sites and LBP-related disability, and other information relevant for the critical appraisal. We contacted two study authors by e-mail as additional information was required regarding missing data to calculate prevalence (see Table 3 for details).

### Risk of bias assessment

We used a modified version of the Risk of Bias Tool for Prevalence Studies to assess the risk of bias [47] (see Additional file 3 for details). For the purpose of this systematic review, the tool was slightly modified; in item 1, the wording was changed from “Was the study’s target population a close representation of the national population in relation to relevant variables?” to “Was the study population representative of the target population?” whereby randomly selected or consecutively selected samples were appraised as low risk of bias and convenience samples as high risk of bias. Furthermore, item 6 was defined for LBP only and we left the example in item 7 open with regards to which study instrument that was used apart from that it must have been validated. This modified risk of bias tool was piloted to ensure that reviewers were consistent in their appraisal. Two reviewer pairs independently performed the risk of bias assessment (CKØ + JH and TFC + MLF). The overall risk of bias (i.e., low, moderate, or high) was determined for each included article based on the reviewers’ consensus, given the responses to the items in this tool. With low overall risk of bias further research is very unlikely to change the confidence in the estimate, moderate overall risk of bias indicate that further research is likely to have an important impact on our confidence in the estimate and may change the estimate, and high overall risk of bias imply that further research is very likely to have an important impact on our confidence in the estimate and is likely to change the estimate. All authors were involved in the final assessment of risk of bias. The GRADE approach was not used in this review for overall appraisal of the quality of the evidence due to lack of guidance for systematic reviews on prevalence data using this methodology [48].

### Data synthesis and analysis

The results of the literature search, risk of bias assessment and data extraction are summarized in tables and figures. The proportion of participants with persistent LBP and co-occurring musculoskeletal pain are described as prevalences and subgrouped according to study population, distribution and location(s) of co-occurring pain. The precision of the prevalences was assessed with 95% confidence intervals (CI) calculated using the exact method in Stata (StataCorp, College Station, Texas, USA). Differences in age- and sex-specific prevalences and distribution pattern of co-occurring musculoskeletal pain are described but not pooled as few studies stratified on these factors. Furthermore, we were not able to assess LBP-related disability or possible differences between working versus general populations as intended, due to the limited reporting in the included articles.

## Results

### Search results and study selection

**Fig. 1:**
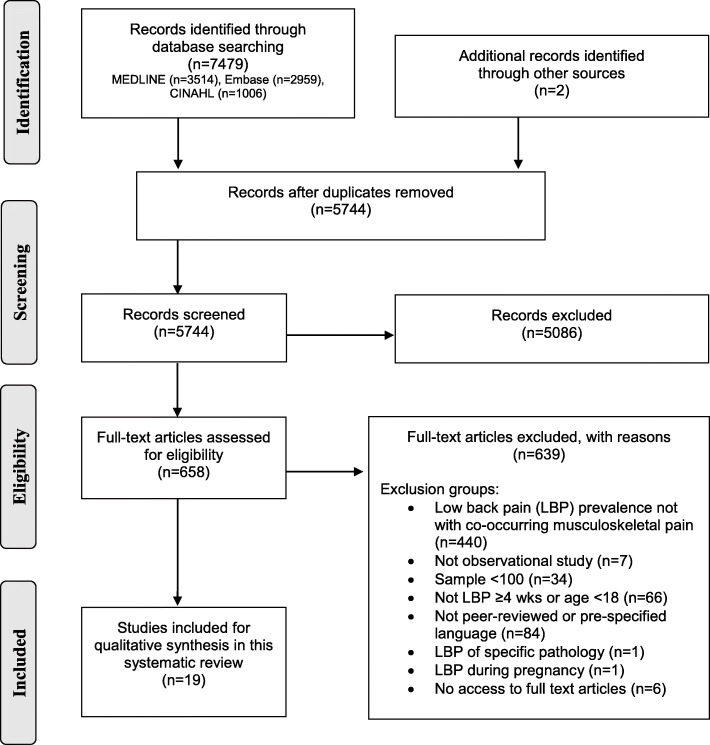
PRISMA Flow chart of literature search and study selection

The study selection processes are illustrated in Fig. 1. A total of 7481 articles were identified through the literature searches and through other sources. After removal of duplicates, 5744 articles were screened at title/abstract level and a total of 658 articles were screened at carried forward for full-text screening. Finally, 19 articles were considered eligible for this systematic review [28–46].

### Study characteristics

The main characteristics of the included articles are presented in Table 1. Ten articles were based on data from the general population, two articles reported on working populations and seven articles reported on clinical populations. Study designs were cross-sectional (*n*=11), prospective (*n*=5), and retrospective (*n*=3); for prospective and retrospective studies, baseline data were considered. The articles were published between 1998 and 2019, while the data were collected between 1985 and 2017 (not reported in two articles). All the included articles were published in English, apart from one in Norwegian [37]. The articles originated from the Scandinavian countries (*n*=7), other European countries (*n*=5), USA (*n*=4) and Japan (*n*=3). A total of 34,492 individuals with persistent LBP and co-occurring musculoskeletal pain were included in our evidence synthesis. Participation rate varied from 19.4 to 100% in different studies while number of people analysed with persistent LBP varied from 100 to 7523. Age was reported both as mean, frequencies within age categories and quartiles, preventing us from reporting an overall mean age; however, most studies included middle-aged and older people (40 to ≥75 years of age). The proportion of females ranged from 48 to 68%, apart from the study with a working population that only included females. Ten of the 19 articles collected prevalence data with questionnaires (five with own questionnaire), and nine used a combination of questionnaires, interviews, and physical examination.

**Table 1:** Main characteristics of the 19 included articles grouped by study population

1st Author Year Country	Study design Year of study (baseline)	Sample size n invited (n at baseline [%]) {n analysed}^**a**^	Age (yrs) and sex^**a**^ mean [SD], age category ***n*** [%], quartile Q2 [Q1, Q3] (♀ ***n*** [%])		Method for assessing prevalence of persistent LBP and co-occurring musculoskeletal pain
**General population**					
Jiménez-Trujillo 2019 [37] Spain	Cross-sectional (2014)	approx. 37500^b^ (22,321 [59.5]^b^) {5189}	♀ 18 to 34; 203 [6.3]^c^ 35 to 54; 888 [27.3]^c^ 55 to 74; 1265 [38.9]^c^ ≥75; 894 [27.5]^c^	♂ 18 to 34; 133 [6.9]^c^ 35 to 54; 723 [37.3]^c^ 55 to 74; 745 [38.4]^c^ ≥75; 338 [17.4]^c^	Questionnaire and Interview (EHISS)
			(3250 [62.6])		
Fujii 2018 [34] Japan	Cross-sectional (2015)	270,000 (52,353 [19.4]) {3100}	44.5 [11.2] (1483 [48.0])		Questionnaire (own)
Takahashi 2018 [47] Japan	Cross-sectional (2011 to 2012)	34,802 (14,364 [41.3]) {1378}^d^	♀ < 50; 101 [15.1]^c^ 50 to 59; 158 [23.6]^c^ 60 to 69; 263 [39.3]^c^ ≥70; 147 [22.0]^c^	♂ < 50; 128 [18.0]^c^ 50 to 59; 172 [24.3]^c^ 60 to 69; 287 [40.5]^c^ ≥70; 122 [17.2]^c^	Questionnaire (own)
			(669 [48.5])		
Nordstoga 2017 [43] Norway	Prospective cohort (1995 to 1997)	93,898 (65,237 [69.5]) {7523}	50.3 [12.0]^b^ (4484 [59.6])		Questionnaire (adapted SNQ)
Kamada 2014 [38] Japan	Cross-sectional (2009)	6000 (4559 [76.0]) {605}	62.8 [10.6] (303 [50.1])		Questionnaire (modified KNEST)
Di lorio 2007 [32] Italy	Cross-sectional (1998)	1270 (958 [75.4]) {306}	74,5 [6,6] (209 [68.3])		Interview (own) Physical examination (including SPPB)
Weiner 2003 [48] USA	Cross-sectional (1997 to 1998)	3075 (2766 [90.1]) {208}	73.5 [2.9] (134^a^ [64.4])		Questionnaire (own) Physical examination (EPESE, Health ABC functional capacity scale)
Natvig 2001 [42] Norway	Cross-sectional (1994)	4577^a^ (2893 [63.2]) {531}^e^	43.1 [14.1]^e^ (334 [62.9])		Questionnaire (SNQ)
Kjellman 2001 [39] Sweden	Retrospective cohort (1985)	213 (213 [100]) {100}	40.4 [2.9] (NR)		Questionnaire or Interview (own + diagnostic codes)
Hoddevik 1999 [36] Norway	Cross-sectional (1994 to 1997)	106,244 (67,338 [63.4]) {6422}	40 to 42 yrs (3865 [60.2])		Questionnaire and Interview (own)
**Working population**					
Andersen 2013 [30] Denmark	Prospective cohort (2004 to 2005)	12,744 (9949 [78.1]) {1089}	47.0 [8] (1089 [100])		Questionnaire (SNQ)
Parot-Schinkel 2013 [45] France	Cross-sectional (2002 to 2005)	NR (3710 [approx. 90]) {616}	NR (for target population 38.4 [10.4]) (264 [42.9])		Questionnaire (French version of SNQ)
**Clinical population**					
Rundell 2019 [46] USA	Prospective cohort (2011 to 2013)	13376^b^ (5239 [39.2]^c^) {899}	74.0 [6.7] (613 [68.0])		Interview (own + diagnostic codes)
Herman 2018 [35] USA	Cross-sectional (2016 to 2017)	6342 (2024 [31.9]^c^) {1129}^c^	NR (NR)		Questionnaire (own)
MacLellan 2017 [40] Irland	Retrospective cohort (2011 to 2015)	915 (915 [100]) {416}	44.6 [12.2] (NR)		Interview (own) Physical examination (5 physical performance tests)
Panagopoulos 2014 [44] Denmark	Prospective cohort (2011 to 2012)	5791 (2974 [51.4]) {2974}	51.0 [15] (1546 [52.0])		Questionnaire (own)
Elfving 2009 [33] Sweden	Prospective cohort (NR)	362 (312 [86.2]) {265}	43.0 [NR] (NR)		Questionnaire (own)
Manchikanti 2003 [41] USA	Cross-sectional (NR)	378 (378 [100]) {300}	LBP only: 52.0 [1.3] (83 [55.0])	LBP + NP or TSP: 44.0 [1.1] (104 [69.0])	Interview (own) Physical examination (diagnostic blocks)
Davies 1998 [31] UK	Retrospective cohort (1989 to 1992)	5279 (5279 [100]) {2007}	52.0 [41, 65] 3176 [60.2]^c^		Interview (own recorded on data form incl. 9 body sites from IASP Subcommittee on Taxonomy, 1986)

*Abbreviations*: *EHISS* European Health Interview Survey for Spain, *EPESE* Established Populations for Epidemiologic Studies in the Elderly performance battery for lower extremity function, *KNEST* Knee Pain Screening Tool, *LBP* low back pain, *NP* neck pain, *NR* not reported, *SD* standard deviation, *SNQ* Standardised Nordic Questionnaire, *SPPB* Short Physical Performance Battery, *TSP* thoracic spine pain, *Q1* lower quartile, *Q2* median, *Q3* upper quartile

^a^ With persistent low back pain

^b^ Data not published in paper and hence received after communication with first author or found in cited method paper

^c^ Calculated by authors

^d^ Includes moderate to very severe persistent low back pain (very mild and mild low back pain [*n*=1594] were omitted from analysis)

^e^ Unpublished data provided by first author that includes participants with persistent low back pain ≥8 wks *n*=531 (*n*=120 with localised persistent low back pain, *n*=167 with low back pain + 1–3 additional pain sites, *n*= 244 with low back pain + 4–9 additional pain sites)

The definition of LBP was typically based on location combined with duration, and in some instances with pain intensity. All the included studies relied on information from questionnaires and interviews that in six studies included pain drawings [30, 31, 35, 38, 43, 44]. Five studies used current pain duration (ranging from ≥4 to 8 weeks) as an inclusion criteria [35, 38–40, 42, 43], while 11 studies used accumulated pain duration within the past year (ranging from ≥3 to 6 months) or variations of accumulated ‘daily pain’ during the past year as inclusion criteria [28–34, 36, 37, 41, 45]. One study used ≥90 days of sickness absence due to LBP during a 2-year period as inclusion criteria [44] and another followed IASP Classification of Chronic Pain [46].

### Critical appraisal

The risk of bias assessment is shown in Table 2. In the overall rating, 14 articles were judged to have a low risk of bias [29–35, 38–43, 46], three articles a moderate risk of bias [28, 36, 37], and two articles a high risk of bias [44, 45]. Overall, the internal validity was judged to be slightly better than the external validity, also, when the articles with an overall high risk of bias were excluded. Notably, seven of the 10 articles with general population samples were rated to have a high risk of non-response bias (item 4). The two articles on working populations had a low risk of bias on all items. For the studies on clinical populations with an overall low risk of bias (two high risk of bias studies disregarded), the risk of bias was mainly related to external validity and non-response bias.

**Table 2:**
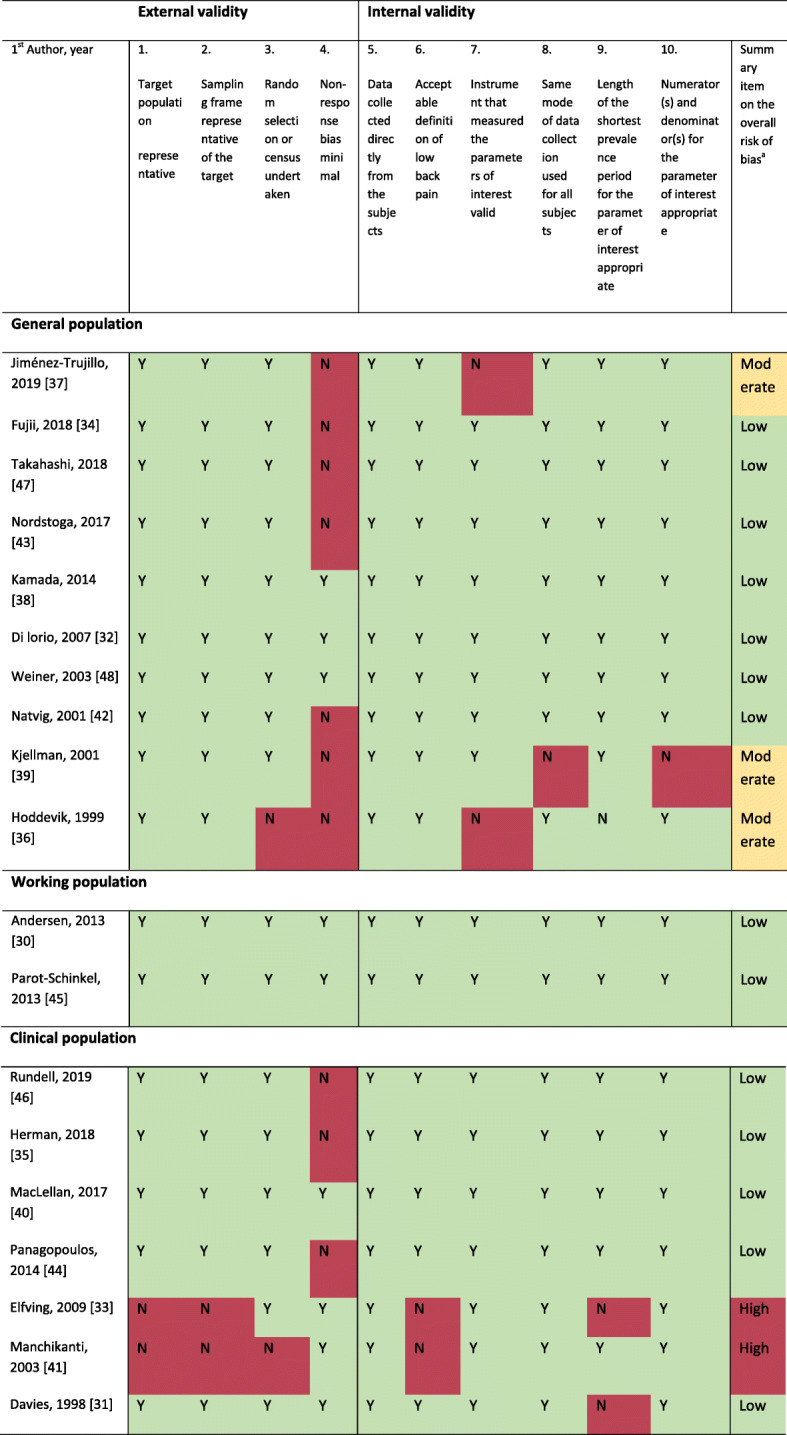
Risk of bias assessment of the 19 included articles grouped by study population (modified from Hoy et al., 2012)

*Abbreviations*: *Y* yes (low risk of bias), *N* no (high risk of bias)

^a^ Summary of overall risk of bias indicated by colour (green = low risk of bias, further research is very unlikely to change our confidence in the estimate; yellow = moderate risk of bias, further research is likely to have an important impact on our confidence in the estimate and may change the estimate; red = high risk of bias, further research is very likely to have an important impact on our confidence in the estimate and is likely to change the estimate).

### Results of individual studies

**Fig. 2:**
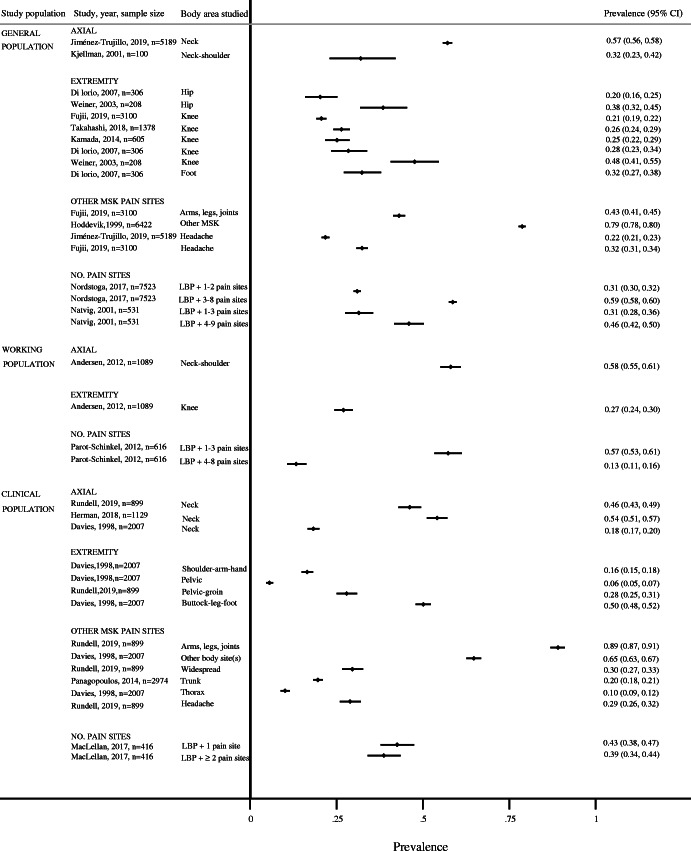
Forest plot of overall summary of the results for the articles with low or moderate risk of bias

Since the included studies were not considered sufficiently homogenous, we chose not to conduct a statistical meta-analysis and the results were therefore synthesised narratively. Table 3 provides a summary of the non-weighted prevalence of co-occurring musculoskeletal pain. Figure 2 gives an overall summary of the results in a forest plot and shows the results for the articles with low or moderate risk of bias. Studies with an overall high risk of bias were not included in the evidence synthesis. Based on the results from the individual articles, co-occurring pain could be grouped into three main categories: i) co-occurring axial pain, ii) co-occurring extremity pain, and iii) other co-occurring multi-site pain (from ≥1 pain site to pain in several body sites).

**Table 3:** Distribution and prevalence of co-occurring musculoskeletal pain among individuals with persistent low back pain grouped by study population

1st Author Year	Axial pain n/N (%) [non-weighted] ♀ / ♂ (if reported)	Extremity pain n/N (%)[non-weighted] ♀ / ♂ (if reported)	Other co-occurring MSK pain sites/no. of pain sites n/N (%)[non-weighted] ♀ / ♂ (if reported)		No. of options for pain sites and regions in addition to LBP
**General population**					
Jiménez-Trujillo 2019 [37]	+ neck: 2963/5189 (57.1) ♀ 2089/2963 (70.5)^a^ ♂ 874/2963 (29.5)^a^		+ headache^d^: 1130/5189 (21.8) ♀ 860/1130 (76.1) ♂ 270/1130 (23.9)		2 (neck, headache)
Fujii 2018 [34]		+ knee: 639/3100 (20.6)	+ headache^d^: 1004/3100 (32.4)	+ arms, legs or joints: 1336/3100 (43.1)	3 (knee, headache, arms/legs/joints)
Takahashi 2018 [47]		+ knee: 364/1378 (26.4)^a, b^			1 (knee)
Nordstoga 2017 [43]			+ 1–2 pain sites: 2331/7523 (31.0)^a^ ♀ 1180/2331 (50.6)^a^ ♂ 1151/2331 (49.4)^a^	+ 3–8 pain sites: 4412/7523 (58.6) ♀ 2978/4412 (67.5)^a^ ♂ 1434/4412 (32.4)^a^	8 (neck, shoulders/ upper arms, elbows, wrists/ hands, upper back, hips, knees, ankles/ feet)
Kamada 2014 [38]		+ knee: 152/ 605 (25.1)^c^			1 (knee)
Di lorio 2007 [32]		+ hip: 62^a^/306 (20.3) + knee: 87^a^/306 (28.4) + foot: 99^a^/306 (32.4)			3 (hip, knee, foot)
Weiner 2003 [48]		+ hip: 80^a^/208 (38.7) + knee: 99^a^/208 (47.6)			2 (hip, knee)
Natvig 2001 [42]			+ 1–3 pain sites: 167/531 (31.5)^c^ ♀100/167 (59.9)^c^ ♂ 67/167 (40.1)^c^	+ 4–9 pain sites (“widespread”): 244/531 (46.0)^c^ ♀ 162/244 (66.4)^c^ ♂ 82/244 (33.6)^c^	9 (head^d^, neck, shoulder, elbow, hand/wrist, upper back, hip, knee or ankle/foot)
Kjellman 2001 [39]	+ neck-shoulder: 32^a^/100 (32.0)				1 (neck/shoulder)
Hoddevik 1999 [36]				+ other MSK pain: 5057/6422 (78.7)^a^ ♀ 3252/5057 (64.3)^a^ ♂ 1805/5057 (35.7)^a^	1 (other MSK)
**Working population**					
Andersen 2013 [30]	+ neck-shoulder: ♀ 632/1089 (58.0)	+ knee: ♀ 294/1089 (27.0)			2 (neck/shoulder, knee)
Parot-Schinkel 2013 [45]			+ 1–3 pain sites: 353/616 (57.3)^a^ ♀ 145/264 (≈ 55)^a^ ♂ 208/352 (≈ 59)^a^	+ 4–8 pain sites: 82/616 (13.3) ♀ 50/264 (≈ 19) ♂ 32/352 (≈ 9)	8 (neck, shoulder/ arm, elbow/forarm, wrist/ hand, upper back, hip/thigh, knee/lower leg, ankle/foot)
**Clinical population**					
Rundell^e^ 2019 [46]	+ neck: 415/899 (46.2)	+ pelvic or groin: 251/899 (27.9)	+ headache^d^: 260/899 (28.9)	+ arms, legs or joints: 801/899 (89.1) + widespread: 266/899 (29.6)	6 (neck, pelvic/groin, headache, stomach, arms/legs/joints, widespread)
Herman 2018 [35]	+ neck: 611/1129 (54.1)^a^				1 (neck)
MacLellan 2017 [40]			+ 1 pain site: 177/416 (42.6)^a^	+ ≥2 pain sites: 161/416 (38.7)^a^	4 (knee, other MSK [not specified which other MSK for those with persistent LBP (i.e. upper- and lower extremity, spinal /headache^d^)]
Panagopoulos 2014 [44]			+ chest-abdomen-groin: 583/2974 (19.6) ♀ 303/1576 (19.2)^a^ ♂ 280/1398 (20.0)^a^		1 (trunk)
Elfving 2009 [33]	+ neck: 43/265 (16.2)^a^ + thoracic: 26/265 (9.8)^a^ + neck and thoracic: 116/265 (43.8)^a^				3 (neck, thoracic, neck and thoracic)
Manchikanti 2003 [41]	+ neck and/or thoracic: 150/300 (50.0)				1 (neck and/or thoracic)
Davies^e^ 1998 [31]	+ neck: 367/2007 (18.3) ♀ 218/367 (59.4) ♂ 149/367 (40.6)	+ shoulder-arm-hand: 331/2007 (16.5) ♀ 199/331 (60.1) ♂ 132/331 (39.9) + pelvic: 112/2007 (5.6) ♀ 71/112 (63.4) ♂ 41/112 (36.6) + buttock-leg-foot: 1006/2007 (50.1) ♀ 595/1006 (59.1) ♂ 411/1006 (40.9)	+ thorax: 203/2007 (10.1) ♀ 118/203 (58.1) ♂ 85/203 (41.9)	+ other body site(s): 1299/2007 (64.7) ♀ 779/1299 (60.0) ♂ 520/1299 (40.0)	6 (neck, shoulder/arm/hand, pelvis, buttock/leg/foot, thorax, other body site(s))

*Abbreviations*: *LBP* low back pain, *NR* not reported, *MSK* musculoskeletal

^a^ Calculated by us

^b^ Among those with moderate, severe and very severe persistent LBP as those with very mild and mild persistent LBP were omitted in paper

^c^ Information provided by the author of the article

^d^ Headache was not part of our search strategy, but we included the prevalences of headache for the otherwise 5 eligible studies where this was reported

^e^ The study by Rundell et al. also included abdominal pain and the study by Davies et al. included both abdominal pain, pain in the head-face-mouth and anal-perineal-genital pain that is not reported

For co-occurring axial pain, the prevalence of neck and neck/shoulder pain ranged from 32 to 57% in the *general population* [28, 36], 58% in the *working population* [38], and from 18 to 54% in *clinical populations* [40, 41, 46].

For co-occurring extremity pain in the hip, knee, and foot the prevalence in the *general population* ranged from 20 to 48% [29, 30, 32–34], while in the *working population* the prevalence of co-occurring knee pain was 27% [38]. In *clinical populations*, the prevalence co-occurring buttock, leg, or foot pain was 50% [46], the prevalence of pelvic and pelvic-groin pain ranged from 6 to 28% [40, 46], and the prevalence of co-occurring shoulder-arm-hand pain was 17% in the only study that considered upper extremity pain [46].

In the *general population* the prevalence of any co-occurring pain in arms, legs, or joints were 43% [29] and other musculoskeletal pain 79% [37], while the prevalence of co-occurring pain in arms, legs, or joints was reported to be 89% and the prevalence of widespread pain (defined as pain in most of your body) was 30% in a *clinical population* [40]. Another clinical population study reported the prevalence of co-occurring chest-abdomen-groin pain (‘anterior trunk pain’) to be 20% [43]; yet another clinical population study reported the prevalence of co-occurring thorax pain to be 10%, and pain in other body sites 65% [46]. Headache was not part of our search strategy, but we have included the prevalences of headache in studies that otherwise was eligible, and it was reported to be 22 to 32% in the *general population* [28, 29] and 29% in a *clinical population* [40]. Additionally, headache was included in two studies that counted number of pain sites [35, 42].

Our results clearly indicate that additional pain sites are common among people with persistent LBP, but reported pain sites are dependent on which sites were asked for in the individual studies. Given a higher number of possible options of co-occurring pain sites, persistent LBP only was reported to be 10–35% across populations (i.e., 10.4% [31]; 18.8% [42]; 21.3% [37]; 22.6% [35]; 29.4% [39]; 35.3% [46]). However, when fewer pain sites were considered the prevalence of persistent LBP only were markedly higher (i.e., 73.6% [30], and 74.9% [32], when the only option was co-occurring knee pain; 45.9% [41] with only co-occurring neck pain as option).

The prevalence of co-occurring neck pain was more than twice as high among females than among males in a general population study that reported on sex differences [28], while for co-occurring neck pain in a clinical population study, about 60% were females [46]. About 30% of those around 75 years reported co-occurring hip pain. Among those reporting co-occurring knee pain, the prevalence tended to increase with age (from 21% at age 45 up to 48% at age 74). Furthermore, the sex distribution among those reporting fewer additional pain sites was similar, while a higher proportion of those reporting a higher number of pain sites were females [31, 35, 39].

Four articles reported on co-occurring pain in relation to LBP-related disability. In the population-based study by Nordstoga et al. [31], persons who reported 3–8 additional pain sites had 16–27% lower probability of recovery from LBP over a 10–11 years period compared to persons with 1–3 additional pain sites. Similarly, in a clinical population of elderly, the Roland Morris Disability Questionnaire (RMDQ) score increased by 0.65 points (95% CI 0.43 to 0.86) for every additional pain site [40]. Additionally, co-occurring pelvic/groin pain, pain in arms, legs, or joints, and widespread pain were all associated with increased long-term LBP disability [40]. Furthermore, patients with persistent LBP who presented with co-occurring anterior trunk pain had significantly higher disability levels measured by RMDQ than those with localised LBP (adjusted group difference at baseline 2.41 [0.34 to 4.49], at 3 months 3.78 [1.37 to 6.18], at 12 months 2.89 [0.67 to 5.11]), but the presence of this co-occurring pain did not affect the rate of recovery of LBP [43]. Socioeconomic status was sparsely considered in the included articles. Though, MacLellan and co-workers [42] found patients with obesity and two or more additional pain sites (in a group where 67.5% had persistent LBP) to have a higher unemployment rate, being retired or unable to work because of disability, having two or more children, or being female compared with patients without any pain.

## Discussion

To our knowledge, this is the first systematic review to appraise and summarise the evidence on the distribution and prevalence of co-occurring musculoskeletal pain among people with persistent LBP. Nineteen articles met the inclusion criteria of which 17 were considered to have a low/moderate risk of bias with risks mainly related to external validity and non-response bias. Three categories of co-occurring pain were identified: (i) axial pain, (ii) extremity pain, and (iii) multi-site musculoskeletal pain. Across the study populations, about 20–60% of participants (*n*=5020/10413) reported co-occurring axial pain while 6–50% reported co-occurring extremity pain (*n*=3576/9592). Pain in multiple sites co-occurring with persistent LBP appears to be common, but the prevalence depends on how it is investigated. This is reflected in the varying prevalences reported for other co-occurring pain sites (i.e., 10–89%). The inconsistency in the number of response options for pain sites in addition to LBP may have affected our observations by masking actual co-occurring pain patterns. We were not able to draw any firm conclusions regarding the association between co-occurring pain and age, or sex. However, we observed that the majority of studies where co-occurring lower extremity pain was assessed included populations with a higher mean age, and that having more pain sites in addition to persistent LBP was more common among females. LBP-related disability in relation to co-occurring musculoskeletal pain was scantily reported but increasing number of pain sites was reported to reduce probability of recovery and decrease work ability.

Persistent LBP is a common component of multi-site pain [49], chronic pain [50], as well as chronic widespread pain [51, 52]. This is in line with our finding that co-occurring pain is common among individuals with persistent LBP.

Neck pain by itself is prevalent [53], but also commonly co-occurs with LBP [8]. In fact, the two have been suggested to be the same clinical entity [54]. Also, lower extremity pain (i.e., excluding sciatica) has been reported to cluster with back pain [21]. This might be due to the lower extremities’ weightbearing function, and may also be seen in light of development of osteoarthritis, which is more common in lower extremity joints [55], and associated with older age [56]. We observed an up to 20% higher prevalence of hip and knee pain in the two studies from USA [34, 40] compared to the study from Italy [33], which may be related to general higher Body Mass Index in USA [57]. Few studies investigated co-occurring upper extremity pain, which is probably due to the way co-occurring pain was measured, that upper-extremity pain more commonly co-occur with neck pain, or that it is related to specific working populations not included in this review as seen in a study by Haukka and co-workers [58].

A high number of co-occurring pain sites was more common among females than men. This is in line with previous studies [14, 59] and has been explained by, for example, higher vulnerability among females [60], hormonal influence [61] and adverse physical working conditions and mental strain [62]. We were not able to stratify on age, but previous studies have reported LBP, as a central part of multi-site pain, among adolescents [63] and LBP with co-occurring pain sites has been associated with older age [64, 65].

Lastly, we observed decreased workability with increased number of co-occurring pain sites. This is in line with other studies, showing that co-occurring pain is a stronger driver of LBP disability than type of occupation [66]. This highlights the importance of considering co-occurring pain as a distinct risk factor for disability retirement [67, 68].

### Methodological considerations

Strengths of this review include a published protocol with registration in PROSPERO, and adherence to PRISMA recommendations and other guidelines for systematic reviews of prevalence studies [69]. Our search was comprehensive and supervised by an experienced research librarian. Based on information given in the abstracts, it was difficult to assess eligibility. We therefore carried a total of 658 articles forward for full-text screening. This reflects the lack of studies considering persistent LBP in the context of co-occurring musculoskeletal pain and of suitable Medical Subject Headings terms and keywords to cover it. For critical appraisal we used ‘Risk of Bias Tool for Prevalence Studies’ which has been reported to have a high inter-rater agreement [47]. This tool uses overall summary risk of bias based on the rater’s judgment, which conforms with Grades of Recommendation, Assessment, Development and Evaluation (GRADE) and Cochrane approaches, rather than using cut-points from summary scores from numerical rating scales that has been discouraged [70]. We retrospectively restricted the study sample size to improve precision as also recommended by Munn et al. [71]. It is arguably inappropriate to conduct meta-analysis for prevalence studies due to the large heterogeneity and differences within characteristics of study populations, and a qualitative description of these variations across study populations has been encouraged instead [72, 73]. This heterogeneity negatively impact the overall certainty of the evidence.

The generalizability of our results is limited given relatively few studies within each co-occurring musculoskeletal area and population type, particularly working populations. Importantly, all articles originated from high-income countries. We also excluded studies with people reporting LBP of < 4 weeks duration. However, this was a pragmatic decision to, on the one hand, avoid inclusion of very few studies and capture studies including people with fluctuating persistent LBP [74], and on the other hand, avoid inclusion of shorter acute episodes of LBP. Definition of LBP varied among reviewed articles, with commonly data on localisation, duration, and sometime intensity, but none fulfilled the optimal definition of LBP for prevalence studies [75], which indicate that this definition is not well known and that awareness of this could be encouraged among researchers providing prevalence data.

For co-occurring musculoskeletal pain, number of pain sites in addition to LBP ranged from 1 to 9 with limited information on the nature of this co-occurring pain. This may have an impact on the observed patterns because the primary studies did not attempt to map underlying patterns of co-occurring pain sites. The problem with inconsistency in the number of pain sites asked for is also seen in other similar studies with variations from six to 26 pain sites [76–79] and a need for a set number of pain sites has been proposed [51].

### Implications

Persistent LBP is very often accompanied by pain in other body regions and thus part of other pain or disease clusters. The available literature on this is, however, quite varied and therefore there is a need for more uniform means of measuring co-occurring musculoskeletal pain to allow for more robust data on the actual existing patterns and to provide better context and interpretability. Understanding *how* pain occur in patterns and clusters can help inform prognosis as well as clinical research, because certain pain patterns among those with persistent LBP may result in more consequences and require different prevention and treatment strategies [23]. Clinically, patients with LBP and a more complex clinical picture are known to get inadequate care [80–82]. This suggests a need for better guidance in clinical care and a move from single-site guidelines [83] to recommendations that cover multi-care pathways for people with persistent LBP including their concomitant conditions [84]. However, this requires prioritising and investment by health authorities, not only to acquire the knowledge base needed to inform healthcare personnel, but also to empower patients to better self-manage if the goal is to decrease disability and costs [85].

## Conclusions

Co-occurring pain among people with persistent LBP is common. Predominant co-occurring pain sites include axial, extremity and multi-site pain. A high number of co-occurring pain sites was also common, in particular among females, and observed more often with disability, and decreased work ability. In spite of most studies having an overall low risk of bias, there was substantial between-study heterogeneity and inconsistent reporting of co-occurring pain sites in the primary studies and we caution readers when interpreting the results.

## Supplementary Information

**Additional file 1.** PRISMA checklist.

**Additional file 2.** Search strategy.

**Additional file 3.** Risk of Bias Tool (Modified from Hoy D. et al. [25]).

## Data Availability

All the data generated and analysed during this study are included in this published article and its Additional files.

## Abbreviations

LBP: Low back pain
PRISMA: Preferred Reporting Items for Systematic Reviews and Meta-Analysis
CKØ: Cecilie K. Øverås
MSJ: Melker S. Johansson
TFC: Tarcisio F. de Campos
BN: Bård Natvig
JH: Jan Hartvigsen
MLF: Manuela L. Ferreira
PJM: Paul Jarle Mork
IASP: International Association for the Study of Pain
RMDQ: Roland Morris Disability Questionnaire
GRADE: Grades of Recommendation, Assessment, Development and Evaluation

## References

[1] Hartvigsen J, Hancock MJ, Kongsted A, Louw Q, Ferreira ML, Genevay S (2018). What low back pain is and why we need to pay attention. Lancet..

[2] Dunn KM, Campbell P, Jordan KP (2013). Long-term trajectories of back pain: cohort study with 7-year follow-up. BMJ Open.

[3] Meucci RD, Fassa AG, Faria NM. Prevalence of chronic low back pain: systematic review. Rev Saude Publica. 2015;49. 10.1590/S0034-8910.2015049005874. 10.1590/S0034-8910.2015049005874 PMC4603263 26487293

[4] Duffield SJ, Ellis BM, Goodson N, Walker-Bone K, Conaghan PG, Margham T (2017). The contribution of musculoskeletal disorders in multimorbidity: implications for practice and policy. Best Pract Res Clin Rheumatol.

[5] Gore M, Sadosky A, Stacey BR, Tai KS, Leslie D (2012). The burden of chronic low back pain: clinical comorbidities, treatment patterns, and health care costs in usual care settings. Spine (Phila Pa 1976).

[6] Hagen EM, Svensen E, Eriksen HR, Ihlebaek CM, Ursin H (2006). Comorbid subjective health complaints in low back pain. Spine (Phila Pa 1976).

[7] Skarpsno ES, Mork PJ, Nilsen TIL, Nordstoga AL (2020). Influence of sleep problems and co-occurring musculoskeletal pain on long-term prognosis of chronic low back pain: the HUNT study. J Epidemiol Community Health.

[8] Hartvigsen J, Davidsen M, Hestbaek L, Sogaard K, Roos EM (2013). Patterns of musculoskeletal pain in the population: a latent class analysis using a nationally representative interviewer-based survey of 4817 Danes. Eur J Pain.

[9] Hartvigsen J, Natvig B, Ferreira M (2013). Is it all about a pain in the back?. Best Pract Res Clin Rheumatol.

[10] Kamaleri Y, Natvig B, Ihlebaek CM, Benth JS, Bruusgaard D (2008). Number of pain sites is associated with demographic, lifestyle, and health-related factors in the general population. Eur J Pain.

[11] Macfarlane GJ (1999). Generalized pain, fibromyalgia and regional pain: an epidemiological view. Baillieres Best Pract Res Clin Rheumatol.

[12] Yabe Y, Hagiwara Y, Sekiguchi T, Sugawara Y, Tsuchiya M, Yoshida S (2020). Musculoskeletal pain in other body sites is associated with new-onset low back pain: a longitudinal study among survivors of the great East Japan earthquake. BMC Musculoskelet Disord.

[13] Carnes D, Parsons S, Ashby D, Breen A, Foster NE, Pincus T (2007). Chronic musculoskeletal pain rarely presents in a single body site: results from a UK population study. Rheumatology (Oxford).

[14] Coggon D, Ntani G, Walker-Bone K, Palmer KT, Felli VE, Harari R (2017). Epidemiological differences between localized and nonlocalized low back pain. Spine..

[15] Gerhardt A, Eich W, Janke S, Leisner S, Treede RD, Tesarz J (2016). Chronic widespread back pain is distinct from chronic local back pain: evidence from quantitative sensory testing, pain drawings, and psychometrics. Clin J Pain.

[16] Artus M, Campbell P, Mallen CD, Dunn KM, van der Windt DA (2017). Generic prognostic factors for musculoskeletal pain in primary care: a systematic review. BMJ Open.

[17] Bayattork M, Jakobsen MD, Sundstrup E, Seidi F, Bay H, Andersen LL (2019). Musculoskeletal pain in multiple body sites and work ability in the general working population: cross-sectional study among 10,000 wage earners. Scand J Pain.

[18] Burgess R, Mansell G, Bishop A, Lewis M, Hill J (2020). Predictors of functional outcome in musculoskeletal healthcare: an umbrella review. Eur J Pain.

[19] Blyth FM, Van Der Windt DA, Croft PR (2015). Chronic disabling pain: a significant public health problem. Am J Prev Med.

[20] Kamaleri Y, Natvig B, Ihlebaek CM, Bruusgaard D (2008). Localized or widespread musculoskeletal pain: does it matter?. Pain..

[21] Schmidt CO, Baumeister SE (2007). Simple patterns behind complex spatial pain reporting? Assessing a classification of multisite pain reporting in the general population. Pain..

[22] Meisingset I, Vasseljen O, Vøllestad NK, Robinson HS, Woodhouse A, Engebretsen KB, et al. Novel approach towards musculoskeletal phenotypes. Eur J Pain. 2020. 10.1002/ejp.1541. 10.1002/ejp.1541 32040225

[23] Øverås CK, Johansson MS, de Campos TF, Ferreira ML, Natvig B, Mork PJ (2017). Prevalence and pattern of co-occurring musculoskeletal pain and its association with back-related disability among people with persistent low back pain: protocol for a systematic review and meta-analysis. Syst Rev.

[24] Moher D, Liberati A, Tetzlaff J, Altman DG (2009). Preferred reporting items for systematic reviews and meta-analyses: the PRISMA statement. PLoS Med.

[25] Hartling L, Featherstone R, Nuspl M, Shave K, Dryden DM, Vandermeer B (2016). The contribution of databases to the results of systematic reviews: a cross-sectional study. BMC Med Res Methodol.

[26] Hartling L, Featherstone R, Nuspl M, Shave K, Dryden DM, Vandermeer B (2017). Grey literature in systematic reviews: a cross-sectional study of the contribution of non-English reports, unpublished studies and dissertations to the results of meta-analyses in child-relevant reviews. BMC Med Res Methodol.

[27] Bramer WM, Milic J, Mast F (2017). Reviewing retrieved references for inclusion in systematic reviews using EndNote. J Med Libr Assoc.

[28] Hoy D, Brooks P, Woolf A, Blyth F, March L, Bain C (2012). Assessing risk of bias in prevalence studies: modification of an existing tool and evidence of interrater agreement. J Clin Epidemiol.

[29] Borges Migliavaca C, Stein C, Colpani V, Barker TH, Munn Z, Falavigna M (2020). How are systematic reviews of prevalence conducted? A methodological study. BMC Med Res Methodol.

[30] Andersen LL, Clausen T, Persson R, Holtermann A (2013). Perceived physical exertion during healthcare work and risk of chronic pain in different body regions: prospective cohort study. Int Arch Occup Environ Health.

[31] Davies HT, Crombie IK, Macrae WA (1998). Where does it hurt? Describing the body locations of chronic pain. Eur J Pain.

[32] Di Iorio A, Abate M, Guralnik JM, Bandinelli S, Cecchi F, Cherubini A (2007). From chronic low back pain to disability, a multifactorial mediated pathway: the InCHIANTI study. Spine..

[33] Elfving B, Asell M, Ropponen A, Alexanderson K (2009). What factors predict full or partial return to work among sickness absentees with spinal pain participating in rehabilitation?. Disabil Rehabil.

[34] Fujii T, Oka H, Katsuhira J, Tonosu J, Kasahara S, Tanaka S (2018). Association between somatic symptom burden and health-related quality of life in people with chronic low back pain. PLoS One.

[35] Herman PM, Kommareddi M, Sorbero ME, Rutter CM, Hays RD, Hilton LG (2018). Characteristics of chiropractic patients being treated for chronic low back and neck pain. J Manip Physiol Ther.

[36] Hoddevik GH, Selmer R (1999). Chronic low back pain in 40-year olds in 12 Norwegian counties. Tidsskr Nor Laegeforen.

[37] Jiménez-Trujillo I, López-de-Andrés A, Del Barrio JL, Hernández-Barrera V, Valero-de-Bernabé M, Jiménez-García R (2019). Gender differences in the prevalence and characteristics of pain in Spain: report from a population-based study. Pain Med.

[38] Kamada M, Kitayuguchi J, Lee IM, Hamano T, Imamura F, Inoue S (2014). Relationship between physical activity and chronic musculoskeletal pain among community-dwelling Japanese adults. J Epidemiol.

[39] Kjellman G, Oberg B, Hensing G, Alexanderson K (2001). A 12-year follow-up of subjects initially sicklisted with neck/shoulder or low back diagnoses. Physiother Res Int.

[40] MacLellan G, Dunlevy C, Blake C, Breen C, Gaynor K, E OM (2017). Musculoskeletal pain profile of obese individuals attending a multidisciplinary weight management service. Obes Facts.

[41] Manchikanti L, Hirsch JA, Pampati V (2003). Chronic low back pain of facet (zygapophysial) joint origin: is there a difference based on involvement of single or multiple spinal regions?. Pain Physician.

[42] Natvig B, Bruusgaard D, Eriksen W (2001). Localized low back pain and low back pain as part of widespread musculoskeletal pain: two different disorders? A cross-sectional population study. J Rehabil Med.

[43] Nordstoga AL, Nilsen TIL, Vasseljen O, Unsgaard-Tondel M, Mork PJ (2017). The influence of multisite pain and psychological comorbidity on prognosis of chronic low back pain: longitudinal data from the Norwegian HUNT study. BMJ Open.

[44] Panagopoulos J, Hancock MJ, Kongsted A, Hush J, Kent P (2014). Does anterior trunk pain predict a different course of recovery in chronic low back pain?. Pain..

[45] Parot-Schinkel E, Descatha A, Ha C, Petit A, Leclerc A, Roquelaure Y (2012). Prevalence of multisite musculoskeletal symptoms: a French cross-sectional working population-based study. BMC Musculoskelet Disord.

[46] Rundell SD, Patel KV, Krook MA, Heagerty PJ, Suri P, Friedly JL (2019). Multi-site pain is associated with long-term patient-reported outcomes in older adults with persistent back pain. Pain Med.

[47] Takahashi A, Kitamura K, Watanabe Y, Kobayashi R, Saito T, Takachi R (2018). Epidemiological profiles of chronic low back and knee pain in middle-aged and elderly Japanese from the Murakami cohort. J Pain Res.

[48] Weiner DK, Haggerty CL, Kritchevsky SB, Harris T, Simonsick EM, Nevitt M (2003). How does low back pain impact physical function in independent, well-functioning older adults? Evidence from the health ABC cohort and implications for the future. Pain Med.

[49] Croft P (2016). Picking targets in fog: the pluses and minuses of defining chronic widespread pain. Pain..

[50] Fayaz A, Croft P, Langford RM, Donaldson LJ, Jones GT (2016). Prevalence of chronic pain in the UK: a systematic review and meta-analysis of population studies. BMJ Open.

[51] Andrews P, Steultjens M, Riskowski J (2018). Chronic widespread pain prevalence in the general population: a systematic review. Eur J Pain.

[52] Mansfield KE, Sim J, Jordan JL, Jordan KP (2016). A systematic review and meta-analysis of the prevalence of chronic widespread pain in the general population. Pain..

[53] Safiri S, Kolahi AA, Hoy D, Buchbinder R, Mansournia MA, Bettampadi D (2020). Global, regional, and national burden of neck pain in the general population, 1990-2017: systematic analysis of the global burden of disease study 2017. Bmj..

[54] Leboeuf-Yde C, Fejer R, Nielsen J, Kyvik KO, Hartvigsen J (2012). Pain in the three spinal regions: the same disorder? Data from a population-based sample of 34,902 Danish adults. Chiropr Man Therap.

[55] Johnson VL, Hunter DJ (2014). The epidemiology of osteoarthritis. Best Pract Res Clin Rheumatol.

[56] Vina ER, Kwoh CK (2018). Epidemiology of osteoarthritis: literature update. Curr Opin Rheumatol.

[57] Collaboration NRF (2016). Trends in adult body-mass index in 200 countries from 1975 to 2014: a pooled analysis of 1698 population-based measurement studies with 19·2 million participants. Lancet..

[58] Haukka E, Leino-Arjas P, Solovieva S, Ranta R, Viikari-Juntura E, Riihimäki H (2006). Co-occurrence of musculoskeletal pain among female kitchen workers. Int Arch Occup Environ Health.

[59] Hagen K, Linde M, Heuch I, Stovner LJ, Zwart JA (2011). Increasing prevalence of chronic musculoskeletal complaints. A large 11-year follow-up in the general population (HUNT 2 and 3). Pain Med.

[60] Pagé MG, Fortier M, Ware MA, Choinière M (2018). As if one pain problem was not enough: prevalence and patterns of coexisting chronic pain conditions and their impact on treatment outcomes. J Pain Res.

[61] Wáng YX, Wáng JQ, Káplár Z (2016). Increased low back pain prevalence in females than in males after menopause age: evidences based on synthetic literature review. Quant Imaging Med Surg.

[62] Neupane S, Leino-Arjas P, Nygård CH, Oakman J, Virtanen P (2017). Developmental pathways of multisite musculoskeletal pain: what is the influence of physical and psychosocial working conditions?. Occup Environ Med.

[63] Holden S, Rathleff MS, Roos EM, Jensen MB, Pourbordbari N, Graven-Nielsen T (2018). Pain patterns during adolescence can be grouped into four pain classes with distinct profiles: a study on a population based cohort of 2953 adolescents. Eur J Pain.

[64] Machado LAC, Viana JU, da Silva SLA, Couto FGP, Mendes LP, Ferreira PH (2018). Correlates of a recent history of disabling low back pain in community-dwelling older persons: the pain in the elderly (PAINEL) study. Clin J Pain.

[65] Patel KV, Guralnik JM, Dansie EJ, Turk DC (2013). Prevalence and impact of pain among older adults in the United States: findings from the 2011 national health and aging trends study. Pain..

[66] Coggon D (2019). Prevention of musculoskeletal disability in working populations: the CUPID study. Occup Med (Lond).

[67] Haukka E, Kaila-Kangas L, Ojajarvi A, Miranda H, Karppinen J, Viikari-Juntura E (2013). Pain in multiple sites and sickness absence trajectories: a prospective study among Finns. Pain..

[68] Neupane S, Nygård CH, Prakash KC, von Bonsdorff MB, von Bonsdorff ME, Seitsamo J (2018). Multisite musculoskeletal pain trajectories from midlife to old age: a 28-year follow-up of municipal employees. Occup Environ Med.

[69] Hoffmann F, Eggers D, Pieper D, Zeeb H, Allers K (2020). An observational study found large methodological heterogeneity in systematic reviews addressing prevalence and cumulative incidence. J Clin Epidemiol.

[70] O'Connor SR, Tully MA, Ryan B, Bradley JM, Baxter GD, McDonough SM (2015). Failure of a numerical quality assessment scale to identify potential risk of bias in a systematic review: a comparison study. BMC Res Notes.

[71] Munn Z, Moola S, Riitano D, Lisy K (2014). The development of a critical appraisal tool for use in systematic reviews addressing questions of prevalence. Int J Health Policy Manag.

[72] Munn Z, Moola S, Lisy K, Riitano D, Tufanaru C (2015). Methodological guidance for systematic reviews of observational epidemiological studies reporting prevalence and cumulative incidence data. Int J Evid Based Healthc.

[73] Saha S, Chant D, McGrath J (2008). Meta-analyses of the incidence and prevalence of schizophrenia: conceptual and methodological issues. Int J Methods Psychiatr Res.

[74] Kongsted A, Hestbaek L, Kent P (2017). How can latent trajectories of back pain be translated into defined subgroups?. BMC Musculoskelet Disord.

[75] Dionne CE, Dunn KM, Croft PR, Nachemson AL, Buchbinder R, Walker BF (2008). A consensus approach toward the standardization of back pain definitions for use in prevalence studies. Spine (Phila Pa 1976).

[76] Coggon D, Ntani G, Palmer KT, Felli VE, Harari R, Barrero LH (2013). Patterns of multisite pain and associations with risk factors. Pain..

[77] de Luca K, Wong A, Eklund A, Fernandez M, Byles JE, Parkinson L (2019). Multisite joint pain in older Australian women is associated with poorer psychosocial health and greater medication use. Chiropr Man Therap..

[78] Gerdle B, Björk J, Cöster L, Henriksson K, Henriksson C, Bengtsson A (2008). Prevalence of widespread pain and associations with work status: a population study. BMC Musculoskelet Disord.

[79] Hunt IM, Silman AJ, Benjamin S, McBeth J, Macfarlane GJ (1999). The prevalence and associated features of chronic widespread pain in the community using the ‘Manchester’ definition of chronic widespread pain. Rheumatology (Oxford).

[80] Croft P, Sharma S, Foster NE (2020). Primary care for low back pain: we don’t know the half of it. Pain..

[81] Panagioti M, Geraghty K, Johnson J, Zhou A, Panagopoulou E, Chew-Graham C (2018). Association between physician burnout and patient safety, professionalism, and patient satisfaction: a systematic review and meta-analysis. JAMA Intern Med.

[82] Ramanathan S, Hibbert P, Wiles L, Maher CG, Runciman W (2018). What is the association between the presence of comorbidities and the appropriateness of care for low back pain? A population-based medical record review study. BMC Musculoskelet Disord.

[83] Caneiro JP, Roos EM, Barton CJ, O'Sullivan K, Kent P, Lin I (2020). It is time to move beyond 'body region silos' to manage musculoskeletal pain: five actions to change clinical practice. Br J Sports Med.

[84] Lowe DB, Taylor MJ, Hill SJ (2017). Associations between multimorbidity and additional burden for working-age adults with specific forms of musculoskeletal conditions: a cross-sectional study. BMC Musculoskelet Disord.

[85] Veasley C, Clare D, Clauw DJ, Cowley T, Nguyen RHN, Reinecke P, Vernon SD, Williams DA. Impact of chronic overlapping pain conditions on public health and the urgent need for safe and effective treatment: 2015 analysis and policy recommendations. Chronic Pain Res Alliance. Published May 2015. http://www.chronicpainresearch.org/public/CPRA_WhitePaper_2015-FINAL-Digital.pdf. Accessed 4 Mar 2020.

