# Supplementary material for: Distribution and prevalence of musculoskeletal pain co-occurring with persistent low back pain: a systematic review
Source: BMC Musculoskelet Disord. 2021 Jan 18;22:91. doi: 10.1186/s12891-020-03893-z (PMC7814622; doi:10.1186/s12891-020-03893-z)
Supplement: Supplementary file 1 — Additional file 1. PRISMA checklist. [file 12891_2020_3893_MOESM1_ESM.docx]

**Table 1** Main characteristics of the 19 included articles grouped by study population

| **1^st^ Author**  **Year**  **Country** | **Study design**  **Year of study**  **(baseline)** | **Sample size**  **n invited**  **(n at baseline [%])**  **{n analysed}^a^** | **Age (yrs) and sex^a^**  **mean [SD], age category n [%], quartile Q2 [Q1, Q3]**  **(♀ n [%])** | | **Methdod for assessing prevalence of persistent LBP and co-occuring musculoskeletal pain** | |
| --- | --- | --- | --- | --- | --- | --- |
| **General population** | | | | | | |
| Jiménez-Trujillo  2019 [37]  Spain | Cross-sectional  (2014) | approx. 37500^b^  (22321 [59.5]^b^)  {5189} | ♀ 18 to 34; 203 [6.3]^c^  35 to 54; 888 [27.3]^c^  55 to 74; 1265 [38.9]^c^  ≥75 ; 894 [27.5]^c^ | ♂ 18 to 34; 133 [6.9]^c^  35 to 54; 723 [37.3]^c^  55 to 74; 745 [38.4]^c^  ≥75 ; 338 [17.4]^c^ | Questionnaire and Interview (EHISS) | |
|  |  |  | (3250 [62.6]) | |  |  |
| Fujii  2018 [34]  Japan | Cross-sectional  (2015) | 270000  (52353 [19.4])  {3100} | 44.5 [11.2]  (1483 [48.0]) |  | Questionnaire (own) | |
| Takahashi  2018 [47]  Japan | Cross-sectional  (2011 to 2012) | 34802  (14364 [41.3])  {1378}^d^ | ♀ <50; 101 [15.1]^c^  50 to 59; 158 [23.6]^c^  60 to 69; 263 [39.3]^c^  ≥70; 147 [22.0]^c^ | ♂ <50; 128 [18.0]^c^  50 to 59; 172 [24.3]^c^  60 to 69; 287 [40.5]^c^  ≥70; 122 [17.2]^c^ | Questionnaire (own) | |
|  |  |  | (669 [48.5]) |  |  |  |
| Nordstoga  2017 [43]  Norway | Prospective cohort  (1995 to 1997) | 93898  (65237 [69.5])  {7523} | 50.3 [12.0]^b^  (4484 [59.6]) | | Questionnaire (adapted SNQ) | |
| Kamada  2014 [38]  Japan | Cross-sectional  (2009) | 6000  (4559 [76.0])  {605} | 62.8 [10.6]  (303 [50.1]) | | Questionnaire (modified KNEST) | |
| Di lorio  2007 [32]  Italy | Cross-sectional  (1998) | 1270  (958 [75.4])  {306} | 74,5 [6,6]  (209 [68.3]) | | Interview (own)  Physical examination (including SPPB) | |
| Weiner  2003 [48]  USA | Cross-sectional  (1997 to 1998) | 3075  (2766 [90.1])  {208} | 73.5 [2.9]  (134^a^ [64.4]) | | Questionnaire (own)  Physical examination (EPESE, Health ABC functional capacity scale) | |
| Natvig  2001 [42]  Norway | Cross-sectional  (1994) | 4577^a^  (2893 [63.2])  {531}^e^ | 43.1 [14.1]^e^  (334 [62.9]) | | Questionnaire (SNQ) | |
| Kjellman  2001 [39]  Sweden | Retrospective cohort  (1985) | 213  (213 [100])  {100} | 40.4 [2.9]  (NR) | | Questionnaire or Interview (own + diagnostic codes) | |
| Hoddevik  1999 [36]  Norway | Cross-sectional  (1994 to 1997) | 106244  (67338 [63.4])  {6422} | 40 to 42 yrs  (3865 [60.2]) | | Questionnaire and Interview (own) | |
| **Working population** | | | | | | |
| Andersen  2013 [30]  Denmark | Prospective cohort  (2004 to 2005) | 12744  (9949 [78.1])  {1089} | 47.0 [8]  (1089 [100]) | | Questionnaire (SNQ) | |
| Parot-Schinkel  2013 [45]  France | Cross-sectional  (2002 to 2005) | NR  (3710 [approx. 90])  {616} | NR (for target population 38.4 [10.4])  (264 [42.9]) | | Questionnaire (French version of SNQ) | |
| **Clinical population** | | | | | | |
| Rundell  2019 [46]  USA | Prospective cohort  (2011 to 2013) | 13376^b^  (5239 [39.2]^c^)  {899} | 74.0 [6.7]  (613 [68.0]) | | | Interview (own + diagnostic codes) |
| Herman  2018 [35]  USA | Cross-sectional  (2016 to 2017) | 6342  (2024 [31.9]^c^)  {1129}^c^ | NR  (NR) | | | Questionnaire (own) |
| MacLellan  2017 [40]  Irland | Retrospective cohort  (2011 to 2015) | 915  (915 [100])  {416} | 44.6 [12.2]  (NR) | | | Interview (own)  Physical examination (5 physical performance tests) |
| Panagopoulos 2014 [44]  Denmark | Prospective cohort  (2011 to 2012) | 5791  (2974 [51.4])  {2974} | 51.0 [15]  (1546 [52.0]) | | | Questionnaire (own) |
| Elfving  2009 [33]  Sweden | Prospective cohort  (NR) | 362  (312 [86.2])  {265} | 43.0 [NR]  (NR) | | | Questionnaire (own) |
| Manchikanti  2003 [41]  USA | Cross-sectional  (NR) | 378  (378 [100])  {300} | LBP only:  52.0 [1.3]  (83 [55.0]) | LBP + NP or TSP:  44.0 [1.1]  (104 [69.0]) | | Interview (own)  Physical examination (diagnostic blocks) |
| Davies  1998 [31]  UK | Retrospective cohort  (1989 to 1992) | 5279  (5279 [100])  {2007} | 52.0 [41, 65]  3176 [60.2]^c^ |  | | Interview (own recorded on data form incl. 9 body sites from IASP Subcommittee on Taxonomy, 1986) |

Abbreviations: EHISS, European Health Interview Survey for Spain; EPESE, Established Populations for Epidemiologic Studies in the Elderly performance battery for lower extremity function; KNEST, Knee Pain Screening Tool; LBP, low back pain; NP, neck pain; NR, not reported; SD, standard deviation; SNQ, Standardised Nordic Questionnaire; SPPB Short Physical Performance Battery;TSP, thoracic spine pain; Q1, lower quartile; Q2, median; Q3, upper quartile.

^a^ With persistent low back pain

^b^ Data not published in paper and hence received after communication with first author or found in cited method paper

^c^ Calculated by authors

^d^ Includes moderate to very severe persistent low back pain (very mild and mild low back pain [n=1594] were omitted from analysis)

^e^ Unpublished data provided by first author that includes participants with persistent low back pain ≥8 wks n=531 (n=120 with localised persistent low back pain, n=167 with low back pain + 1-3 additional pain sites, n= 244 with low back pain + 4-9 additional pain sites).
