# Supplementary material for: Distribution and prevalence of musculoskeletal pain co-occurring with persistent low back pain: a systematic review
Source: BMC Musculoskelet Disord. 2021 Jan 18;22:91. doi: 10.1186/s12891-020-03893-z (PMC7814622; doi:10.1186/s12891-020-03893-z)
Supplement: Supplementary file 2 — Additional file 2. Search strategy. [file 12891_2020_3893_MOESM2_ESM.docx]

**Table 2** Risk of bias assessment of the 19 included articles grouped by study population (modified from Hoy et al., 2012)

|  | **External validity** | | | | **Internal validity** | | | | | |  |
| --- | --- | --- | --- | --- | --- | --- | --- | --- | --- | --- | --- |
| 1^st^ Author, year | 1.  Target population  representative | 2.  Sampling frame representative of the target | 3.  Random selection or census undertaken | 4.  Non-response bias minimal | 5.  Data collected directly from the subjects | 6.  Acceptable definition of low back pain | 7.  Instrument that measured the parameters of interest valid | 8.  Same mode of data collection used for all subjects | 9.  Length of the shortest prevalence period for the parameter of interest appropriate | 10.  Numerator(s) and denominator(s) for the parameter of interest appropriate | Summary item on the overall risk of bias^a^ |
| **General population** | | | | | | | | | | | |
| Jiménez-Trujillo, 2019 [37] | Y | Y | Y | N | Y | Y | N | Y | Y | Y | Moderate |
| Fujii, 2018 [34] | Y | Y | Y | N | Y | Y | Y | Y | Y | Y | Low |
| Takahashi, 2018 [47] | Y | Y | Y | N | Y | Y | Y | Y | Y | Y | Low |
| Nordstoga, 2017 [43] | Y | Y | Y | N | Y | Y | Y | Y | Y | Y | Low |
| Kamada, 2014 [38] | Y | Y | Y | Y | Y | Y | Y | Y | Y | Y | Low |
| Di lorio, 2007 [32] | Y | Y | Y | Y | Y | Y | Y | Y | Y | Y | Low |
| Weiner, 2003 [48] | Y | Y | Y | Y | Y | Y | Y | Y | Y | Y | Low |
| Natvig, 2001 [42] | Y | Y | Y | N | Y | Y | Y | Y | Y | Y | Low |
| Kjellman, 2001 [39] | Y | Y | Y | N | Y | Y | Y | N | Y | N | Moderate |
| Hoddevik, 1999 [36] | Y | Y | N | N | Y | Y | N | Y | N | Y | Moderate |
| **Working population** | | | | | | | | | | | |
| Andersen, 2013 [30] | Y | Y | Y | Y | Y | Y | Y | Y | Y | Y | Low |
| Parot-Schinkel, 2013 [45] | Y | Y | Y | Y | Y | Y | Y | Y | Y | Y | Low |
| **Clinical population** | | | | | | | | | | | |
| Rundell, 2019 [46] | Y | Y | Y | N | Y | Y | Y | Y | Y | Y | Low |
| Herman, 2018 [35] | Y | Y | Y | N | Y | Y | Y | Y | Y | Y | Low |
| MacLellan, 2017 [40] | Y | Y | Y | Y | Y | Y | Y | Y | Y | Y | Low |
| Panagopoulos, 2014 [44] | Y | Y | Y | N | Y | Y | Y | Y | Y | Y | Low |
| Elfving, 2009 [33] | N | N | Y | Y | Y | N | Y | Y | N | Y | High |
| Manchikanti, 2003 [41] | N | N | N | Y | Y | N | Y | Y | Y | Y | High |
| Davies, 1998 [31] | Y | Y | Y | Y | Y | Y | Y | Y | N | Y | Low |

Abbreviations: Y, yes (low risk of bias); N, no (high risk of bias)

^a^ Summary of overall risk of bias indicated by colour (green = low risk of bias, further research is very unlikely to change our confidence in the estimate; yellow = moderate risk of bias, further research is likely to have an important impact on our confidence in the estimate and may change the estimate; red = high risk of bias, further research is very likely to have an important impact on our confidence in the estimate and is likely to change the estimate).
