# Supplementary material for: Distribution and prevalence of musculoskeletal pain co-occurring with persistent low back pain: a systematic review
Source: BMC Musculoskelet Disord. 2021 Jan 18;22:91. doi: 10.1186/s12891-020-03893-z (PMC7814622; doi:10.1186/s12891-020-03893-z)
Supplement: Supplementary file 3 — Additional file 3. Risk of Bias Tool (Modified from Hoy D. et al. [25]). [file 12891_2020_3893_MOESM3_ESM.docx]

**Table 3** Distribution and prevalence of co-occurring musculoskeletal pain among individuals with persistent low back pain grouped by study population

| **1^st^ Author**  **Year** | **Axial pain**  **n/N (%) [non-weighted]**  **♀ / ♂ (if reported)** | **Extremity pain**  **n/N (%)[non-weighted]**  **♀ / ♂ (if reported)** | **Other co-occurring MSK pain sites/no. of pain sites**  **n/N (%)[non-weighted]**  **♀ / ♂ (if reported)** | | **No. of options for pain sites and regions in addition to LBP** |
| --- | --- | --- | --- | --- | --- |
| **General population** | | | | | |
| Jiménez-Trujillo  2019  [37] | + neck:  2963/5189 (57.1)  ♀ 2089/2963 (70.5)^a^  ♂ 874/2963 (29.5)^a^ |  | + headache^d^:  1130/5189 (21.8)  ♀ 860/1130 (76.1)  ♂ 270/1130 (23.9) |  | 2  (neck, headache) |
| Fujii  2018 [34] |  | + knee:  639/3100 (20.6) | + headache^d^:  1004/3100 (32.4) | + arms, legs or joints:  1336/3100 (43.1) | 3  (knee, headache, arms/legs/joints) |
| Takahashi 2018 [47] |  | + knee:  364/1378 (26.4)^a, b^ |  |  | 1  (knee) |
| Nordstoga  2017  [43] |  |  | + 1-2 pain sites:  2331/7523 (31.0)^a^  ♀ 1180/2331 (50.6)^a^  ♂ 1151/2331 (49.4)^a^ | + 3-8 pain sites:  4412/7523 (58.6)  ♀ 2978/4412 (67.5)^a^  ♂ 1434/4412 (32.4)^a^ | 8  (neck, shoulders/ upper arms, elbows, wrists/ hands, upper back, hips, knees, ankles/ feet) |
| Kamada  2014 [38] |  | + knee:  152/ 605 (25.1)^c^ |  | | 1  (knee) |
| Di lorio  2007  [32] |  | + hip:  62^a^/306 (20.3)  + knee:  87^a^/306 (28.4)  + foot:  99^a^/306 (32.4) |  | | 3  (hip, knee, foot) |
| Weiner  2003  [48] |  | + hip:  80^a^/208 (38.7)  + knee:  99^a^/208 (47.6) |  | | 2  (hip, knee) |
| Natvig  2001  [42] |  |  | + 1-3 pain sites:  167/531 (31.5)^c^  ♀100/167 (59.9)^c^  ♂ 67/167 (40.1)^c^ | + 4-9 pain sites (“widespread”):  244/531 (46.0)^c^  ♀ 162/244 (66.4)^c^  ♂ 82/244 (33.6)^c^ | 9  (head^d^, neck, shoulder, elbow, hand/wrist, upper back, hip, knee or ankle/foot) |
| Kjellman  2001 [39] | + neck-shoulder:  32^a^/100 (32.0) |  |  | | 1  (neck/shoulder) |
| Hoddevik  1999 [36] |  |  |  | + other MSK pain:  5057/6422 (78.7)^a^  ♀ 3252/5057 (64.3)^a^  ♂ 1805/5057 (35.7)^a^ | 1  (other MSK) |
| **Working population** | | | | | |
| Andersen  2013 [30] | + neck-shoulder:  ♀ 632/1089 (58.0) | + knee:  ♀ 294/1089 (27.0) |  | | 2  (neck/shoulder, knee) |
| Parot-Schinkel 2013 [45] |  |  | + 1-3 pain sites:  353/616 (57.3)^a^  ♀ 145/264 (≈ 55)^a^  ♂ 208/352 (≈ 59)^a^ | + 4-8 pain sites:  82/616 (13.3)  ♀ 50/264 (≈ 19)  ♂ 32/352 (≈ 9) | 8  (neck, shoulder/ arm, elbow/forarm, wrist/ hand, upper back, hip/thigh, knee/lower leg, ankle/foot) |
| **Clinical population** | | | | | |
| Rundell^e^  2019 [46] | + neck:  415/899 (46.2) | + pelvic or groin:  251/899 (27.9) | + headache^d^:  260/899 (28.9) | + arms, legs or joints:  801/899 (89.1)  + widespread:  266/899 (29.6) | 6  (neck, pelvic/groin, headache, stomach, arms/legs/joints, widespread) |
| Herman  2018 [35] | + neck:  611/1129 (54.1)^a^ |  |  |  | 1  (neck) |
| MacLellan  2017 [40] |  |  | + 1 pain site:  177/416 (42.6)^a^ | + ≥2 pain sites:  161/416 (38.7)^a^ | 4  (knee, other MSK [not specified which other MSK for those with persistent LBP (i.e. upper- and lower extremity, spinal /headache^d^)] |
| Panagopoulos  2014 [44] |  |  | + chest-abdomen-groin:  583/2974 (19.6)  ♀ 303/1576 (19.2)^a^  ♂ 280/1398 (20.0)^a^ | | 1  (trunk) |
| Elfving  2009  [33] | + neck:  43/265 (16.2)^a^  + thoracic:  26/265 (9.8)^a^  + neck and thoracic:  116/265 (43.8)^a^ |  |  | | 3  (neck, thoracic, neck and thoracic) |
| Manchikanti  2003 [41] | + neck and/or thoracic:  150/300 (50.0) |  |  | | 1  (neck and/or thoracic) |
| Davies^e^  1998 [31] | + neck:  367/2007 (18.3)  ♀ 218/367 (59.4)  ♂ 149/367 (40.6) | + shoulder-arm-hand:  331/2007 (16.5)  ♀ 199/331 (60.1)  ♂ 132/331 (39.9)  + pelvic:  112/2007 (5.6)  ♀ 71/112 (63.4)  ♂ 41/112 (36.6)  + buttock-leg-foot:  1006/2007 (50.1)  ♀ 595/1006 (59.1)  ♂ 411/1006 (40.9) | + thorax:  203/2007 (10.1)  ♀ 118/203 (58.1)  ♂ 85/203 (41.9) | + other body site(s):  1299/2007 (64.7)  ♀ 779/1299 (60.0)  ♂ 520/1299 (40.0) | 6  (neck, shoulder/arm/hand, pelvis, buttock/leg/foot, thorax, other body site(s)) |

Abbreviations: LBP, low back pain; NR, not reported; MSK, musculoskeletal.

^a^ Calculated by us.

^b^ Among those with moderate, severe and very severe persistent LBP as those with very mild and mild persistent LBP were omitted in paper.

^c^ Information provided by the author of the article.

^d^ Headache was not part of our search strategy, but we included the prevalences of headache for the otherwise 5 eligible studies where this was reported.

^e^ The study by Rundell et al. also included abdominal pain and the study by Davies et al. included both abdominal pain, pain in the head-face-mouth and anal-perineal-genital pain that is not reported.
